# It’s About the Journey – Capturing Stories of the Fluctuating Experiences of Youth Kidney Transplant Patients

**DOI:** 10.1145/3653704

**Published:** 2024-04-26

**Authors:** JULIA C. DUNBAR, WANDA PRATT, EMILY BASCOM, CARA CURRIER, JOSEPH WILLIAM TAN GARCIA, JODI SMITH, JAIME SNYDER, ARI H POLLACK

**Affiliations:** University of Washington, Information School, USA; University of Washington, Information School, USA; University of Washington, Human Centered Design and Engineering, USA; Washington State University, Elson S Floyd School of Medicine, USA; University of Washington, Information School, USA; Seattle Children’s Hospital, Division of Nephrology & University of Washington, School of Medicine, Department of Pediatrics, USA; University of Washington, Information School, USA; Seattle Children’s Hospital, Division of Nephrology & University of Washington, School of Medicine, Department of Pediatrics & University of Washington, Information School, USA

**Keywords:** pediatric kidney transplantation, chronic illness journey, quality of life, storytelling

## Abstract

Youth who undergo a kidney transplant can experience a fluctuation of successes and challenges throughout their chronic illness journey. Designing to capture their journey could help youth to reflect on their experiences, collaborate on their care, and be empowered to live their lives to the fullest. We interviewed 11 youth kidney transplant patients and 12 caregivers to elicit their transplant journey experiences. We found that probing participants about specific parts of their transplant journey gave them structure to tell us rich stories about their experiences. Based on our findings, we discuss informing the design of a tool to support the capturing of stories for youth with chronic illnesses. Designing such tool could help youth and their caregivers to identify barriers, support reflection, and promote self-efficacy. Youth with chronic illnesses already have to change so many aspects of their lives to accommodate their illness, however, by giving them a platform to capture their chronic illness journey, it could encourage them to take more control of their lives and better collaborate with others.

## 1 INTRODUCTION

When youth are diagnosed with a chronic illness, they and their families are thrust into a new, rapidly changing reality. The families’ focus must drastically shift from the routine daily activities of childhood to a new and unfamiliar landscape of medical appointments, medications, and unclear outcomes. Yet, the original activities, needs, and dreams of these youth continue despite their new health challenges. Having a chronic illness as a child adds a new level of complexity, layering a variety of different biological, social, and organizational elements. Successfully interweaving these many different components together can be difficult for both youth and their caregivers, resulting in conflicts between youths’ healthcare and non-healthcare life needs (in short life needs). For example, a youth may struggle to take their medications on time or at all (i.e. a healthcare need), as it can interfere with their social relationships (i.e. feeling awkward having to take their medications in front of their peers) or activities (i.e. playing sports or video games). These conflicts can have a significant impact on their health and well being and require that youth adjust to this new reality of living with a chronic illness. Youth and their families will begin to adjust to their new state of normalcy [80] as their healthcare and life needs begin to harmonize.

In this study, we have worked with youth who have received a kidney transplant, due to kidney disease, a life-threatening chronic illness [15, 30, 84, 91]. While many youth after receiving a kidney transplant eventually get to a point where they feel normal again [113], most struggle on their journey towards normalcy (i.e. when their healthcare and life needs feel at harmony). As a result, Adolescents and Young adults (AYAs) who have received a kidney transplant often struggle to take their required post-transplant medications on time, a major cause of kidney rejection and premature kidney failure [18, 20, 50, 117]. The journey after transplant is not uniform and linear, but instead fluctuates between times where youth struggle with their chronic illness and other times where they successfully integrate their healthcare needs with their life needs [22].

Understanding, and ultimately addressing the conflicts between the healthcare and life needs that affect youths’ normalcy requires a collaborative and comprehensive view of a youth’s journey living with a chronic illness. In the past decade there has been a push in medical, CSCW, and HCI fields towards more patient-centered care [28, 77] as well as understanding patients’ chronic illness journeys [65, 67, 89], but even with this push there is still a need to further explore the intricacies of chronic illness journeys for youth to support them in better understanding their holistic journey. By capturing youths journeys, it may provide them with the necessary tools they need to reflect and identify challenges and successes, and ultimately support better collaboration with their caregivers and clinical care team for the goal of improved harmony and normalcy.

However, to support such collaborative reflection, youth need a way to capture and review their journeys with others. Stories may provide a valuable source of information for youth kidney transplant patients, their care team, and their caregivers by painting a more detailed and vivid picture of the events or experiences that these youth experience during their transplant journey. In addition, comparing stories over time show the evolution and progression of one’s kidney transplant journey. While researchers within a variety of disciplines have explored the collection of individually curated data, such as personal tracked data [74, 87, 108], this type of data tends to be more quantitative in nature and often doesn’t align or connect with their personal and narrative life experiences. CSCW researchers as well as researchers within other fields have explored mediums where patients may have more direct access to stories, such as online communities [56–58, 83, 88, 97], but these spaces often lack the structure required for people to find relevant stories or specific topics. Therefore, there is a need for new tools to help individuals, especially youth, find stories applicable to their chronic illness journey to make sense of their experiences.

Designing tools to capture youth’s kidney transplant journeys has the potential to help youth, their caregivers, and their care team better collaborate and understand their experiences throughout different points of their transplant journeys. It may also provide all stakeholders with a way to reflect on the intricacies of an individual’s journey and identify times when their healthcare and life needs conflict. In this paper we explore the following research question:

1. How can an existing framework help inform the design of a tool to support the collection of youth and caregivers’ transplant journeys?

Overall, we aim to understand how the design of new tools can help youth kidney transplant patients and their caregivers capture a more comprehensive and holistic view of their chronic illness journey. Having the ability to capture their journey over time may help youth transplant patients to build better collaborations with their caregivers and clinical care team to identify barriers, promote self-efficacy, and eventually live the lives they want to live.

## 2 RELATED WORK

Ongoing research in medical, health informatics, CSCW, and HCI fields continues to explore the difficulty of living with a chronic illness as a youth. In this section we explore related work from each of these domains and highlight some of the different complexities of growing up with a severe chronic illness. We first explore literature that addresses these complexities by trying to support youth through technology designed to help them with their self-management, communication with their caregivers or care team, and more. We then delve into literature that explores outcomes tracking and the need for capturing not only youth’s healthcare trajectories and needs, but also their non-healthcare life goals and needs to provide individuals with a more comprehensive view of their chronic illness journeys. Lastly, we explore how past literature in CSCW and HCI has used stories and discuss how stories can be a valuable source of information if captured in a structured format for these individuals to leverage properly.

### 2.1 The Complexity of Supporting and Designing for Youth with Chronic Illnesses

Living with a severe chronic illness as a youth requires managing many different puzzle pieces to ensure that they receive the best possible care while at the same time trying to enjoy their childhood. A variety of factors that affect youth with a chronic illnesses include their physical growth/development [26], psychosocial development [19, 109, 119], and transitioning to adult-care [6, 9, 106]. Prior CSCW and HCI research has explored how technology supports people living with a chronic illness, with a majority of the work focusing on the needs of adults [4, 59, 63, 82, 92–94]. While, some have begun to explore the complexity of leveraging CSCW and HCI technologies to support youth with a chronic illness better, many opportunities to improve outcomes still exist. Previous CSCW and HCI work with this population has explored how technology can support youth in their transition towards self-management [11], collaboration between youth and their caregivers (e.g., their parents) to help youth transition towards independence [11, 54, 55], and more actively participating in their healthcare as well as communicating with their hospital care team (e.g., their clinicians) [53, 55, 122]. Other CSCW and HCI work has focused on the needs of hospitalized youth, including designing patient portals to best align with patient needs and support engagement in their healthcare [39], identifying opportunities and challenges when designing peer support technology within a hospital setting [38, 40], and developing technology to support youth goal setting and collaboration[122].

The underlying thread of most of this work is to better understand how technology can assist and support youth through their chronic illness experiences so that they can be successful in the long term. For these individuals to be successful, it is important to take into consideration not only how to support their health, but also how to keep them on track for the lives they want to live. While some of the work above does consider how to harmonize a youth’s various healthcare and life needs, gaps remain in better understanding, capturing, and identifying barriers and conflicts that occur throughout youth’s chronic illness journeys.

### 2.2 The Importance of Capturing a Comprehensive View of the Chronic Illness Journey

Living with a chronic illness has a profound effect on an individual’s quality of life, especially youth [2, 19, 62, 96, 111, 120]. A key challenge in understanding the impact chronic illness has on an individual is identifying and tracking appropriate outcomes. While tracking clinical outcomes such as lab results, growth parameters, or vital signs are key for understanding the biological factors associated with a chronic illness, they do not necessarily reflect or capture the outcomes or needs that matter most to patients and families. There has been an uptick in collecting patient-generated data and patient-reported outcomes (i.e., data tracked by patients themselves) [12, 36, 43, 81] within the past decade, but even though this data is tracked by patients themselves most of the data still track health outcomes and does not necessarily reflect or capture the goals or needs that matter most to patients and families.

However, researchers have recently started exploring other types of outcomes. Researchers who are a part of the Standardised Outcomes in Nephrology (SONG) initiative are working towards developing a core set of outcomes based on shared priorities of different stakeholders e.g., patients, caregivers, clinicians, researchers, and more [61]. As part of their findings, the SONG researchers have identified a high-priority outcome domain for youth who have kidney transplants called ”Life Participation” [69, 70, 72]. They define life participation as, ”the ability to participate in meaningful activities of daily living,” such as going to school, playing sports, or spending time with family and friends [61]. Even though researchers are starting to explore new types of outcomes, such as life participation, there are still gaps to be filled in understanding and exploring the capturing of both healthcare and life needs.

Access to information reflecting patients’ healthcare and life needs may give patients, their caregivers, and their clinicians a more comprehensive perspective of youth’s chronic illness journeys. Some CSCW and HCI researchers have begun to delve into the intricacies of illness journeys, however, a majority of this work has been focused on adults and people living with cancer [44, 65–67]. Nikkhah et al.’s CSCW paper on journey work does include pediatric cancer patients, but their findings discuss caregiving coordination journeys within the pediatric cancer journey [89]. There are still gaps to be addressed within this space to further explore different populations of youth with chronic illnesses, in our case youth who have had kidney transplants due to a severe chronic illness, requiring acute and chronic illness care. Further exploration into this space can also help to gain a better understanding of how to capture information that may directly help youth gain a deeper understanding of how their healthcare and life needs align or conflict throughout their journey. Capturing a more comprehensive view of a youth’s journey may also provide them with the necessary tools to reflect on and identify barriers throughout their journey so they can better collaborate with caregivers and clinical care team. One source of information that is gaining popularity within health contexts, but still requires further exploration with youth patients, are stories, which is the focus of our research.

### 2.3 Capturing Stories to Reflect on Youth’s Chronic Illness Journeys

The use of storytelling, stories, and narratives has been explored in a variety of research fields over the years. Stories are a valuable source of information because they provide details about an individual’s experience as well as they can depict the evolution of an individual’s journey over time. They are also valuable because they can capture things, such as feelings, emotions, and values, which are key to providing information to help contextualize and frame events. In recent years applications such as StoryWorth [110] or Remento [99] have become more mainstream, aiming to preserve loved ones memories, so their stories can be collaboratively shared between family members. Stories have and continue to be a valuable way to communicate information between people and as a research community we continue to explore the value of storytelling amongst different communities and how technology can support the capturing and transferring of stories.

Storytelling has been explored in a variety of different research domains, including the intersection of storytelling and clinical medicine, known as narrative medicine [1, 75, 85, 86, 100, 118]. Narrative medicine has been used to not only support patients but also support clinicians in better understanding patients lived experiences [1, 85, 86, 100], thus illustrating how stories can be used as a collaborative source of information to help understand and reflect on a more holistic view of patients journeys. An example of how stories have been successfully integrated and used to promote patient-centered care within a hospital system is the VA’s My Life, My Story program [85, 100]. In this program veterans participate in interviews and from their interview their “life history” is cultivated, which is then summarized and integrated into the VA healthcare system for clinicians and patients to have access to [1]. This work done with the VA illustrates the value that stories can provide in a healthcare setting. However, there is still work to be done to further explore non-adult experiences, such as youth chronic illness experiences, and how technologies can be designed to capture stories throughout the entirety of a chronic illness journey.

At the intersection of stories and technology, researchers continue to explore a variety of ways in which technology can help elicit, capture, and tell individuals’ stories. Gordon et al. report on an automated story extraction system that involves capturing stories that people tell each other during conversations within the context of knowledge management [34, 35]. Epstein et al. discuss tracking tools and how these tools can better support individuals in telling their story using their data [23]. Pavel et al. delves into the challenges of capturing different and large amounts of data to display to individuals and approach it through a story-inspired paradigm [95]. Other researchers investigate and expand on ways to support storytelling [101], elicit stories [105], and even how to use stories to support goals [103, 104].

Another avenue of exploration is when to capture an individual’s transplant stories (past, present, or future stories). Most of the time when we think of capturing stories, we think about capturing experiences that have already happened or just happened (i.e. past and present stories). However, research done by Hershfield et al. has shown that people have difficulty aligning their current and future selves as one in the same [45, 46, 48, 90]. Hershfield and others explore the idea of future self-continuity, ”the sense of similarity and connection that is felt between one’s current and future selves” [102], mainly within the domain of finance [24, 47, 49] as well as a few other domains [102, 116]. This idea of future self-continuity is important for youth with chronic illnesses because they still have a majority of their life to live and understanding how their current/past decisions and experiences impact their future is critical. Some CSCW and HCI researchers have also explored the concepts of future self-continuity or future-self in a handful of studies, such as in the designs of future-self avatars for learning [114] and nutrition [32] or studying the effects of future self-continuity within VR in delaying immediate gratification [25]. Even though capturing and documenting past stories may help patients to reflect on their past so that they can grow in their future, making active choices in the present to help their future selves can still be difficult for individuals. Encouraging youth to think about their future in alignment with their past may also be an important consideration when encouraging participants to think about their transplant journey.

Stories can also be made up of different forms of data including, but not limited to written words, photos, audio recordings, or a combination of all of the above. Within the context of having a chronic illness, stories about youth’s experiences are more typically spoken via word of mouth from patient to caregiver, patient to care team, or patient to patient. Capturing youth’s stories via written word or images within a format that can help them is less common. However, capturing individuals written stories and images over time could be a valuable source of information for these youth. How to best capture these stories though, so that youth can best leverage them is more challenging. It will be important to provide a structure for capturing youth stories that help them create a comprehensive picture of their chronic illness journey.

Clinical researchers within the past decade have increasingly sought to better understand youth transplant patient’s experiences directly from youth themselves, enhancing our understanding of youth and their families’ kidney experiences [37, 41, 71, 73, 113]. However, there is still a need for a better understanding of youths’ complex kidney transplant journeys. Dunbar et al.’s Kidney Identity framework [22] may provide a good structure to probe participants about specific parts of their transplant journeys. The framework attempts to capture the complexity of the transplant journey and illustrates fluctuations that may occur throughout the journey from diagnosis to after kidney transplantation. In our methods section we will explain further why we chose to use Dunbar et al’s framework to inform our study design and then in the findings we will discuss the resulting stories that were captured.

## 3 METHODS

Our goal for this project was to examine kidney transplant youth and caregivers’ journey experiences and inform the design of a tool to support capturing their journeys. We analyzed findings from semi-structured interviews with 11 youth kidney transplant patients and 12 caregivers, for a total of 23 participants. The findings from the interviews lead to the characterization of different types of stories about participants kidney transplant journey experiences.

### 3.1 Recruitment of Participants

All participants were recruited from a single pediatric transplant center that is part of a large children’s hospital located within the United States. We had access to participants because two members of the study team are also clinicians at the transplant center. Most participants were recruited via convenience sampling by identifying individuals who fit the eligibility criteria and were scheduled for an upcoming transplant clinic visit. After participants were identified based on the clinic schedule, a member of the research team sent a list of the potential participants to a clinician on the study team to confirm the eligibility of the candidates. All participants were then contacted for recruitment either via phone call, patient portal, or email. We recruited 23 total participants, including 11 youth who have had a kidney transplant and 12 caregivers of these youth.

Our eligibility criteria for participants included: (1) had a kidney transplant (2) English-speaking, (3) 7+ years of age, (4) had non-failing kidney function (i.e. a GFR >30 ml/min/1.73m2) [29], and (5) be a minimum of three-months post-transplant. Our youth participants (n=11) ranged in age between 7 – 22 years (median age = 13). More specifically, between the ages of 7–10 we had n = 4 participants, between 11 – 14 we had n = 3, between 15 – 18 we had n = 3, and between 19 – 22 years old we had n = 1. It is not unusual for youth with complex health conditions to continue being treated at children’s hospitals into young adulthood. In this paper participants of all age ranges may be addressed as youth to keep wording consistent throughout. Our youth participants identified with the following genders: Man/Boy (n = 9), Woman/Girl (n = 2), and Transgender (n = 1), while our caregiver participants (n = 12) ranged between the ages of 27 – 58 (median age = 47) and identified with the following gender: Woman/Girl (n = 11) and Not Reported (n = 1) (see Table 1). One caregiver participant did not report any of their demographic information, which is marked as Not Reported in Table 1. Only one participant dropped out of the study after being consented, this youth participant was not included in the youth participant count.

### 3.2 Study Procedures

Before the interviews, all participants completed a consent/assent session with a member of the research team. The first author interviewed each participant (pediatric participants and caregivers separately) in one ~60-minute long semi-structured interview online via Zoom [123]. Youth and caregiver participants conducted their interviews separately, unless the parent or child felt uncomfortable and requested that the caregiver be present for the pediatric participant’s interview. For almost all interviews, youth and caregivers completed their interviews separately and did not have a caregiver step in during the interview, with the exception of one interview when a caregiver stepped in to help their child answer when they were confused. After interviews were completed, participants completed a Redcap [42] demographics survey (Table 1). Participants (youth and caregivers) were each compensated with a $25 digital eGift Card, which they received via email. All study procedures were approved by the hospital’s Institutional Review Board.

### 3.3 Data Collection

In developing our interview guides and analytical approach, we explored literature related to chronic illness journeys [44, 65–67, 89] and youth kidney transplant experiences [37, 41, 71, 73, 113]. While other work has explored aspects that youth kidney transplant patients and their caregivers experience, we felt that Dunbar et al’s framework provided the most comprehensive and holistic view for this specific population, and more importantly focuses on the youth’s unique and important perspective. Therefore, we utilized this framework to inform our interviews with the purpose of diving deeper into participants transplant experiences.

Dunbar et al. state that their framework, ”ties together multiple facets of their (a youth’s) life including clinical, developmental, and social” [22]. This framework details five dimensions which describe common experiences for both youth and their caregivers throughout their (post) kidney transplant journey:

- **Dimension 1:** Telling One’s Story - hiding vs. self-expression,
- **Dimension 2:** Exchanging Information - information consumers vs. information contributors,
- **Dimension 3:** Transitional Management - family management vs. self-management,
- **Dimension 4:** Building Confidence - worry vs. confidence, and
- **Dimension 5:** Normalizing Kidney Transplantation - feeling different vs. feeling similar.

**Fig. 1.**
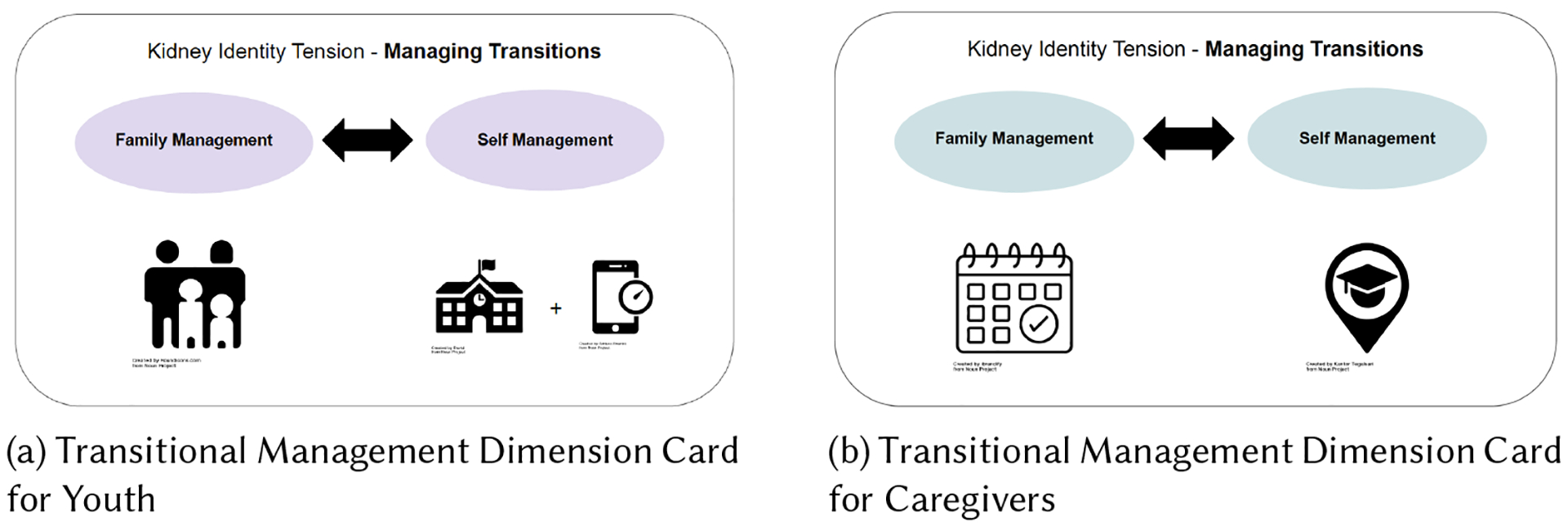
This figure depicts the two Transitional Management Dimension cards displayed to youth and caregivers during their interviews. These cards were simply meant to provide high-level visual representations of each of the five kidney identity dimensions to go along with the scenario descriptions. The card displayed to youth, Figure 1a on the left, shows an info graphic of parents surrounding their two children for family-management as well as two info-graphics of a school + a cell-phone timer for self-management. The card displayed to caregivers, Figure 1b on the right, shows an info graphic of a calendar with a check-mark for family-management and an info graphic location bubble with a college graduation hat in the center of it. This figure uses several icons from creators from the noun project, creator details can be seen on the figure.

Qualitative semi-structured interview guides are often delineative of the topics that the interviewer wishes to explore [16, 17, 68]. Since our research question was, ”*How can an existing framework help inform the design of a tool to support the collection of youth and caregivers’ transplant journeys?”*, we designed our interview guides to reflect Dunbar et al’s framework dimensions to probe participants about their holistic transplant experiences. In particular, we used the five dimensions of the framework to capture the positive and negative fluctuations youth often experience during their transplant journeys and potentially elucidate conflicts between their healthcare and non-health life needs. During the interview, we provided youth and caregiver participants with high-level visual representations of each of the five kidney identity dimensions (see Figure 1a and 1b), via Zoom’s screen-sharing functionality [123]. Alongside the visual representation, we also read out-loud brief example scenarios of each dimension. These scenarios incorporated examples from Dunbar et al’s Kidney Identity findings [22] (see example scenario written below). For example, when describing the Transitional Management Dimension - family management vs. self-management, we read the following two descriptions to youth patients about each side of the dimension:

- **Family Management description:** Griffin is 11 years old and often relies on his parents to remind him about taking his different medications for his kidney. They set timers on his phone for him to follow and also check on him throughout the day to make sure that he takes them.”
- **Self-Management Description:** Griffin is now 14 years old and starting high school. He is excited about starting high school, but also knows he will be involved in after school activities like theatre club and track which is usually when he would take his pills for his kidney. Griffin starts to make plans for the school year and how he will remember to take his medications, such as setting a timer on his phone.

To help explain the Kidney Identity Framework to participants, we used a metaphor, describing it as a pizza, ”*This identity is similar to a pizza, which has different ingredients, such as dough, pizza sauce, cheese, and toppings. Our identity that we will be talking about today has five different ingredients that make up this “kidney identity.”* We used the metaphor to help participants understand the concepts discussed during their interview and allowed them, particularly the youth, to feel comfortable talking about their holistic journey. In addition, we purposefully designed the metaphor as a fun precursor, to relax the participants and help them feel comfortable discussing the different, and at times emotional, aspects of their journey. After this explanation, we began the semi-structured interview questions, organized by dimension. We created separate interview guides for caregivers and youth participants with different example scenarios and descriptions for youth and caregiver participants.

After reading participants the example dimension scenarios, we then moved on and began to ask participants interview questions. The interview questions focused on probing about specific parts of kidney transplant patient’s and caregivers’ journeys to better understand their experiences. All semi-structured interviews were audio and video recorded and later transcribed for analysis.

### 3.4 Data Analysis

To begin analysis, the first and last authors reviewed 3/4 of the transcripts individually and took notes on reoccurring themes that were shared in the interviews individually and subsequently met to discuss and compare observations. Following this initial pass, the first author went through the transcripts a second time and pulled quotes by Kidney Identity dimension, that aligned with the first set of discussed themes. We sorted the quotes by dimension, and then created digital FigJam sticky notes for each quote in FigJam [27]. Next, the research team met and categorized the quotes via affinity diagramming [52, 76]. Each affinity diagramming session (one per Kidney Identity dimension) lasted approximately one to two hours, where two to four people from the research team organized quotes. During each session each person was assigned, reviewed, and sorted a random subset of the quotes independently for 10 to 20 minutes; after individual sorting, the group discussed the individual categories of the quotes. After the group discussion, overlapping categories were identified and grouped together, and finally, re-reviewed all quotes to make sure they were placed in the correct category.

## 4 FINDINGS

We found that probing participants about specific parts of their transplant journey gave them structure to tell us rich stories about their experiences. In this section, we are characterizing the different types of stories that our participants discussed for each of the dimensions of the Kidney Identity framework (see Table 2). Quotes from youth pediatric transplant patients are identified by “Y#” and their primary caregiver as “C#”, with the number being a unique identifier for each participant assigned by the research team.

### 4.1 Dimension 1 – Telling People About Your Kidney Transplant Journey

#### 4.1.1 Stories about Barriers and Benefits to Sharing About Your Journey.

Several participants told us different stories about personal barriers they experienced in telling people about parts of their kidney transplant journey. Y8, Y9, and Y10 were **nervous about how other people would react** when they learned about their transplant journey, while C2 and C4 talked about **feeling unprepared** to explain to people about their child’s illness. Y2 told us a story about the **awkwardness of trying to bring up his looming transplant into everyday conversation** with his friends and now reflecting on the moment, wishing he brought it up sooner. Both Caregivers and youth participants told us stories about the non-linear experiences they have had when trying to open up about the kidney transplant journey. For youth participants having these fluctuating periods of being comfortable talking about their transplant journey was often a part of their **sense-making process**. C5 told us a story about P5’s sense-making process:

“I can definitely relate to this in a sense that his journey’s been on a spectrum where it hasn’t always been the same. He’s gone through periods of what I would call hiding. Where it was a lot of why. Why can’t I? And I think over time, obviously, those questions have all been answered and so he’s just absorbed it and moved on and it has accepted, I think, in his own way, this is my normal.”

(C5)

On the flip-side, Y13 and C13 always felt open to telling people about their kidney transplant journey experiences. Y13 told a story about how their fun-fact during school orientations was about their kidneys, “*It’s like everyone else’s fun facts in sixth grade orientation was like, ”I’m reading this book. I visited this place.” Me, ”I had a kidney transplant when I was three years old, because both of them decided to fail on me and they never figured out why*.” illustrating **acceptance of their illness**. Others like C10 told us how it’s always been a part of Y10’s story because they were born with their illness, “*I mean Y10 was born with kidney issues, so she’s never known any other way*.” Most of the youth and caregivers felt that they fluctuated in their willingness to talk to people about their kidney transplant experiences throughout the course of the transplant journey, however, as both youth and their caregivers reflected on their different periods of “*hiding*” and “*self-expression*” it was transparent that when they had moments of hiding it was often coupled with feeling uncertain, while moments of self-expression were often coupled with feelings of understanding and control over their transplant journey. We can help caregivers and youth reflect on moments of hiding, so they can uncover barriers towards what is making them feel uncertain or in less control of their journey, as well as, seeing when and why they transitioned to moments of self-expression may reveal avenues to help them build towards and sustain staying on the self-expression side of the dimension.

#### 4.1.2 Stories about Community.

When probed about this aspect of their journey, youth participants and their caregivers told us several different types of stories around the category of community. Some caregivers and youth participants told us stories about their immediate family and how the youth’s illness journey has **become a part of their immediate family’s own life as well** (C9, C7, and P9). C9 told us a story about how this has become a part of P9’s grandma’s life and that it affects her day-to-day life:

“She has joined a Facebook group. So, she’s always online, concerned about P9 and thinking about and looking for the latest medicines, treatments for keeping people in remission…So she gives me things that she’s finding in her research…I’ll bring it to X or Dr. X… So, it’s affected her life.”

(C9)

Youth participants and their caregivers talked about how having to **tell others about their kidney transplant journey helped them both build a better community network** as well as it **helped make their experiences more positive**, because they had a community of people with whom to share their stories. C4 told us about how she was encouraged to join a a certain type of pre-school and it led to her telling more people about their (her and her child’s) kidney transplant journey more confidently, “…*And so you create a sense of community*. *And it was really that sense of community that started to empower me to speak up for what I needed to take care of Y4. Like, ”Okay, well he’s a little different and I’m going to practice feeling empowered to say he can’t have this ice cream or whatever it was*.” Even though participants discussed the importance of telling their story and how this can influence their relationship with their community, it was also noted that they lacked the ability to tell their story to other transplant families. The kidney transplant journey can be a long process for these youth and their families if they feel that they do not have the support of a community. Seeing how their journey has affected different parts of their community (e.g., immediate family) it may encourage youth and their caregivers to reflect on their experiences together and overall have more open conversations. Being able to reflect on their successful moments of building their community may also encourage these families to continue building support in moments when they are hesitant to discuss their journey.

### 4.2 Dimension 2 - Exchanging Information Throughout the Transplant Journey

#### 4.2.1 Stories about Lack of Interaction with Other Transplant Families.

When talking about exchanging information, something that we heard from almost all our youth participants was that they have rarely or “…*never talked to someone else who had a transplant*” (Y10). This left some of the youth **feeling alone** in their journey like Y4, “…*usually sometimes (I) feel like I’m the only one who has ever had a kidney transplant as a kid*.” Caregivers also told us stories about feeling alone during stressful times of the kidney transplant journey and like C2 they expressed that it would have been, ”*helpful*” to have heard about other transplant families who have been through similar experiences. A handful of the youth and caregiver participants did have experiences exchanging information with other people who have also been through difficult medical experiences, like Y13 who told us a story about the types of conversations they have with a friend who also frequents the hospital:

“We’ll be at lunch and just normal conversation happening next to us at the table, and we’ll just be like, ”Hey, want to see the MRI I had?” It’s just randomly talking about this stuff to each other, and it’s kind of funny sometimes, when we’re talking about it around other members of our friend group because one of us will share this fact that’s probably horrifying to people who don’t visit the hospital a bunch…”

(Y13)

**Feeling understood** such as Y13 did when they were able to exchange stories with their friend can be beneficial for this community since they often lack interactions with other transplant families. Other potential benefits for this population when sharing their stories with one another could be **exchanging positives about the transplant journey and learning about new medical possibilities**, like C4 who told us about their willingness to talk to others about different medical related things, “…*like how to navigate, and how to advocate, and how to partner with providers, and in the medical system, I’ve always been very open to sharing”*.

Participants also talked about how if they shared stories with another transplant family or patient it may be beneficial, because they could **compare experiences and learn from others across experience levels.** C6 told us about some positive experiences she has had sharing their (her and P6’s) transplant stories online, because they were able to “*compare some notes, compare experiences*”, which gave their family a “*new perspective*”. Hearing about others’ transplant journey stories has the potential to help these patients and their families feel less alone, normalize different points of their journey, hear success stories, learn about new medical possibilities, and overall be able to compare and learn from others across different experience levels.

Even though a majority of both our youth and caregiver participants expressed interest in exchanging information with other transplant families, we did have some participants that were uninterested in connecting with others. These participants lacked interest for several reasons including that they only wanted to experience things on their own (C1), they needed their child to be at a stable enough point of their journey (C7), they were worried people may feel bad when comparing transplant experiences (C7), or they were not at a state of being where they were ready to share (C4). Several participants also expressed hesitation with the idea of connecting online with others, because they “don’t really do social media” (C12), they don’t consider themselves a “computer person” (C8), or they had mixed feelings towards connecting online with others because of past experiences.

#### 4.2.2 Stories about Building Self-Efficacy.

Another theme that emerged as a benefit when exchanging information was the potential for both youth and their caregivers to build self-efficacy. Shifting from receiving to sharing information will not always be a one-way street throughout the transplant journey, instead participants told stories describing how it is **always a learning process**. Y2 and C12 expressed to us how they think that they will always receive and share information:

“Yeah, I think it’s really best to always do kind of both, I would say, to always get information and improve on your own life through that information and also share to help other people.”

(Y2)

“For both of these really, because it’s always and forever, that’s kind of the diagnosis. This is forever. I’m going to have a young adult…I’m going to have to relinquish control, and that’s still going to be hard. Harder in different ways than it will be for my other kids, to do that.”

(C12)

Due to their life long chronic illness, working towards self-efficacy is essential for these youth. In order to work towards self-efficacy it will be important for them to reflect on how they exchange information throughout their transplant journey. Recognizing who, when, and what type of information is important to communicate about throughout their journey is essential in supporting youth as they work towards self-efficacy.

### 4.3 Dimension 3 - Transitional Management Throughout the Transplant Journey

#### 4.3.1 Stories about Responsibility and Planning for the Future.

When probed about the transitional management aspect of their journey, we received a lot of different stories from both youth and their caregivers about **who is responsible for the youth’s kidney health, how they are responsible, and what stage of self-management they are currently in**. In their stories, participants emphasized the notion that transitioning to self-management cannot be done in a single move, but instead it is a learning process for everyone involved and can take many years. Many of our youth participants recognized that they still **rely on their different caregivers** to help manage their kidney health (Y3, Y4, Y5, Y6, Y9, Y10, and Y12). Some caregivers described themselves as the **primary “directors”** (C1) of their child’s kidney health and that they will continue to be in the foreseeable future, because of the child’s current developmental stage.

Youth, as they reached certain stages of their life started to recognize though that they should also start to take on more responsibilities for their kidney health: “*My parents. So, my parents are responsible, but I should be too*…” (Y6). Several of the youth told us stories about the different stages of self-management that they are in, which ranged from drinking water and making sure to go to the bathroom (Y5), to taking their medications on time (Y13, Y8, Y10, Y9, and Y3), and attending doctor’s appointments alone (Y8). However, learning to self-manage is not only about taking medication, but also about learning how to integrate their healthcare into their everyday lives, such as Y13 when they attend a school dance:

“…if I’m going out somewhere with my friends, like to a school dance for example, that I know is going over the 7:30 time period, I’ll tuck my meds into a pocket, bring it along, and I’ll pick one friend… I’ll still have my phone with my alarm in my pocket and everything, but I’ll pick one friend, whoever seems like they’ll be the most responsible at the moment, and just be like, ”Hey, I know I have my alarm for 7:30 to remind me to take my meds, but could you just come find me around that time to make sure I’ve done it?” and I have never missed meds on a school dance night.”

(Y13)

It was clear from several participants stories that as youth get older there is an expectation for them to become more independent, but doing so takes time and often requires parents and youth to start thinking about it from a young age. Such as C10, who told us her current and future thoughts about small steps towards independence for their child, “…*She’s X age though, so she’s a little young to have her own device…So maybe when she’s a little older, we’ll get her a smart watch or something we can send her reminders or I don’t know, something to help her self regulate more…”* (Y10). Caregivers were not the only ones who told us stories about their different goals for transitioning to self-management, several **youths also had different goals for self-efficacy**, like Y5:

“Yeah. I’d like to start taking my medicine on my own and set my own timers and all that…Well, I just sort of I want to be prepared to be more in independent for when I keep growing and eventually get out into the real world. So, yeah, I just want to learn to do things about myself eventually.”

(Y5)

Transitioning from family to self-management can be a tedious learning process for everyone who is involved. Our participants’ stories illustrate that throughout the kidney transplant journey there is an ongoing conversation that must be had, so youth are supported but also encouraged to work towards self-efficacy. Capturing participants fluctuating experiences throughout their journey has the potential to help these families identify what stage of transition they are in, what successes they have had so far, what barriers they are coming up against, recognize if they should start prepping for success, and overall encourage open conversations between youth and their caregivers about who is responsible and how they can work towards their different management goals together.

#### 4.3.2 Stories about Challenges of Transitioning.

Working towards self-management does not come without barriers and the different worries that come along with those barriers. Youth participants of different ages and caregivers talked about different barriers they experience, including but not limited to barriers about: taking medications, drinking enough fluids, communicating with clinicians and other people about their kidney, and more. Youth of all ages and their caregivers recognize that there are different stages to transitioning to self-management and that each step matters. Several caregivers told stories about why they are worried about their kid transitioning to self-management in the future, such as if they will remember to take their medications and schedule appointments (C2). Even when the caregivers were excited to transition their child to self-management, they also had worries in the back of their mind, such as C5:

“Oh yeah. He is definitely ready for that and I’m excited to pass that baton. I think one of our concerns is that…mornings are very hectic and so he takes his meds before he leaves for school, and then before bed. And with the mornings being hectic and he’s not a morning person, so sometimes it’s all he can do to just remember everything to get out the door. And then at bedtime usually he’s just ready to have sort of like free time where he’s not having to follow a schedule or abide by a schedule. So, but I think that, for him, as long as he can put it into memory, he can be very routine. So I don’t think it’ll be a problem.”

(C5)

When talking about this specific aspect of their journey caregivers also brought up stories about how far their child has come and they reflected on **feeling proud of their child**, because of everything they have gone through and the different wins they have experienced along the way. C4 reflected on a story about seeing her son’s personal growth and what that meant for his empowerment, “*I think where this really comes into play a lot is with empowerment in medical situations. Like medical trauma, PTSD, whatever you want to call it, is real for these kiddos. And so that’s really been an evolution of him being able to take control of those situations and make them feel a little bit more manageable, like with blood draws. So in that sense, he’s been very hands-on. Yeah. And wanted to put the little rubber band on, and he chooses his own vein, and chooses his arm, so in that sense. But although that’s kind of a different question, but he does show real, like he wants to be a part of that process*.” By capturing these different wins and providing the ability for both youth and their caregivers to reflect on these different “wins” it can help provide them with a sense of positivity as well as encourage future growth towards self-management.

### 4.4 Dimension 4 - Building Confidence Throughout the Transplant Journey

#### 4.4.1 Stories about Fear.

Interestingly, when discussing the building confidence dimension most of the caregivers we interviewed had stories on the “worried” side of the dimension, but there were almost no quotes from youth participants about that side of the dimension. Each of the caregivers’ stories all stemmed from different types of fears, such as **fear of different kidney related health concerns or fear of the unknown.** One acute fear that several caregivers reflected on was the day of their child’s transplant (C1, C6, and C8). C1 told the story about Y1’s day of transplant and her fear of complications, “*The biggest worry… Especially I was thinking on the day when Y1 gets to be admitted at the operation room, that something happened, because they told me that there might be complications or that he might need blood transfusion or something like that*.” Part of the worry described by C1 is fear of the unknown, which was also a common fear amongst several other caregivers (C2, C3, C9, and C12).

Another fear that was expressed by some of the caregivers was the fear for their child’s well-being when they were in environments not controlled by the caregiver. Even after youth received their transplants several caregivers as well as a youth participant told stories about the fear **of being in an environment where they have less control, and their kidney might become damaged.** Such as C1 who worries when Y1 is at school, “*The only thing that worries me is that*, *during the day when he is at school…I don’t want him to get hurt or to fall, or somebody kicks his stomach or something like that*,” or Y5 who worries when they do different activities, “*So, well, the only thing I really worry about is damaging my kidney if I do activities that are too crazy, and like I get into some accident*”. Beyond fears about health concerns some caregivers discussed their fears of **collateral damage to their child’s life**, such as C12:

“And then within the first few years after the transplant, I’d say maybe between two and four years. The worry is, you’re like, ”*Okay. So my kid has this limitation, but he needs to feel the limitation so that he’s safe, but not be limited in his life*.”

(C12)

Even though caregivers told us stories about the many different types of fears they have for their child, they also told us stories about how their and their child’s confidence has changed throughout the transplant journey.

#### 4.4.2 Stories about the Road to Confidence.

When telling stories about why they feel confident both youth and their caregivers talked about how they feel **confidence in living through the journey.** Y2 told us about how setting a kidney related goal and living through that increased his confidence, “…*because when you have milestones, maybe like getting to one year past transplant and having actually done it, that kind of boosts your confidence, because you look like, ”Wow, I actually been able to do this for an entire year*.” C6 similarly talked about living through P6’s kidney transplant journey:

“…but the confidence I think comes from a lot of just living through it. The trial and error, figuring out what work and keeping positive…It doesn’t have to be a bad thing that you have kidney disease, and I truly believe that he’s the person he is and the person he’s becoming because of it.”

(C6)

Others told stories about feeling more confident, because they were **getting back to their routine and feeling normal again.** Y9 and C9 both told stories about going back to their normal eating habits and not worrying about the type of food Y9 has to eat, “…*having to drink more and like what I eat, for like sodium and potassium and stuff. Now I don’t have to worry as much*” (Y9). While others like Y8 talked generally about “*doing the stuff*” they couldn’t do before “*without any problem*” now.

Both youth and their caregivers also told stories about **confidence in their self-efficacy**. Caregivers like C1 talked about confidence in being able to take care of their child, “…*make me feel confident is that I have the ability to do what is best for my child*”. Youth like Y10 talked about gaining more confidence in things like taking their medications, “*For me medicine, I’m actually kind of confident because I don’t spit it out anymore*.” Our participants seemed to enjoy talking about all the ways they felt confident, because as they talked about it, it gave them the ability to reflect on how strong they are for everything they have been through, prove to themselves that they are able to get back to their version of “normal” as well as show them how they have gained self-efficacy throughout the journey.

### 4.5 Dimension 5 - Normalizing the Transplant Journey

#### 4.5.1 Stories about Removing Healthcare Burdens and Reconnecting with Your Community.

Youth and caregiver participants told us stories about their fluctuation of experiences related to normalizing their journey by establishing a new or comfortable routine. They told us stories about a variety of different healthcare burdens and how their lives have changed by having to deal with these burdens less or not at all post-transplant. Several participants brought up stories about the reduction of burdens when their child was no longer on dialysis, such as C9 who told us about how the everyday task of showering became easier when P9 was off dialysis:

“That was a big issue. ”Mom, I want to take a shower today.” Hold on. Let me get everything ready. Tucking in the wires for dialysis…then you feel similar to everyone else in the family that doesn’t have to put an Aquaguard on to take a shower and be careful and that was a big one.”

(C9)

For patients who have chronic kidney issues what they eat and if they feel good enough to eat can be severely impacted prior to transplant. Several participants including C1, C3, Y3, C9, and C13 all told stories about what it was like for them or their child to be **able to eat more freely.** Others told stories about how their burdens felt less when: they were able to go back home post-transplant, they frequented the hospital less, they didn’t have to go in for labs as much, or when some of their everyday activities became physically easier.

Another aspect that was made easier by having reduced healthcare burdens, which helped participants feel back to their version of normal, was when they were able to **reconnect with their different communities.** C1, C5, and C12 all told stories about how pre-transplant their families had to change their routines and that as a family they couldn’t get out as easily, even weekly activities like going to the “*grocery store*” (C12) was difficult. As opposed to post-transplant, some participants, like C10 expressed how they were able to get out more as a family, “…*we didn’t have to say, ”Okay, we have to go now because Y10 got to do her dialysis.” So, we had a little more wiggle room as far as goes, being able to do things in the evening, that sort of thing*.”

A few caregivers expressed that their children post-transplant started to **rejoin the curve**, such that their kids were catching up on different developmental milestones (e.g., with school). This was mentioned less frequently by caregivers, but for the caregivers who did mention it they were excited that their child was able to reconnect with their similar age-mates. A handful of participants also talked about reconnecting with some of their different communities because they were able to **participate in more physical activities**.

#### 4.5.2 Stories about Accepting Differences and Establishing a “New Normal”.

Another part of normalizing their transplant journey for both youth participants and their caregivers was when they **accepted what made them feel different or established their “new” norms**. Some participants told us how different parts of maintaining their or their child’s kidney health became their **“new normal” routines** throughout different parts of the transplant journey. C5 expressed how dialysis “*started to become normal for us*” *and they went on “autopilot*”. Y8 “…*just felt normal*” a few years into taking their medications and seeing doctors regularly. C6 expressed how they are very intentional about “…*getting into the routine, making it the new normal*”.

Beyond establishing new normal routines, another part of normalizing the transplant journey for participants was accepting their differences as their version of normal. Y13 told us about how they have gotten used to talking about their kidney and answering different questions about it and that they have “…*heard these questions enough times, they just get bored at this point*…”. Other youth participants like Y2 and Y8 expressed that some parts of their kidney transplant journey used to be difficult for them to accept but now they “…*don’t really care anymore*”. It is inevitable that both youth and their caregivers during different parts of their transplant journey will feel “different” but buy bringing into focus what makes them feel their version of “normal” throughout both the difficult and stable parts of their journeys may be key to helping them discover barriers they may be coming up against, feeling a sense of community, and establishing what they want their “normal” to be.

## 5 DISCUSSION

The kidney transplant journey, similar to other chronic illness journeys, consists of many stories that encompass patients’ experiences. Patients’ stories provide youth, caregivers, and clinicians with a holistic view of patients’ transplant journeys. In our findings, we characterized the different stories youth and their caregivers told us when probed about different dimensions of their kidney transplant experiences. We found that using Dunbar et al’s Kidney Identity framework to inform our interview guides helped our participants to reflect and tell us stories about their transplant experiences. Youth in the age ranges we worked with are not always the most forthright in speaking about their experiences, but using the framework helped to ground them at a high-level in the interview topics as well as be more open about their experiences. In the following sections, we will discuss how our findings can inform the design of a kidney journey tool to support the collection of kidney transplant journeys. We demonstrate how designing to capture kidney transplant stories may help youth patients, their caregivers, and their clinicians collaborate to identify barriers, support reflection, and promote self-efficacy for youth kidney transplant patients.

### 5.1 Supporting Reflection

Youth kidney transplant patients, caregivers, and clinicians regularly reflect on youth patients health records and other quantitative data to determine a youth’s health status throughout the transplant journey; however, their ability to contextualize what is happening during these different periods is lacking. After using an existing framework to successfully probe youth transplant patients and their caregivers about their kidney transplant experiences, our findings revealed, that youth and caregivers have a multitude of stories to tell (e.g., stories about building self-efficacy or stories about accepting differences and establishing a ”new normal”). Based on these findings, we conclude that designing a tool to capture the stories of youth kidney transplant patients and their caregivers, can help support stakeholders to reflect on their kidney transplant journeys. Researchers have and continue to explore how technology can play a role in encouraging reflection [5, 13, 112]. Lim et al. [78] has explored the facilitation of self-reflection among individuals with chronic conditions and [8] has designed tools to support health and wellness reflection.

We discovered that using the Kidney Identity framework and its five dimensions to inform our interview guides gave participants structure to reflect on specific aspects of their experiences, while still allowing them to share about a myriad of their experiences as stories. As participants shared their experiences for each of the “Kidney Identity” dimensions we noticed how participants without prompting discussed stories from different times of their transplant journey, including their past, their present, and even their future. This population of youth in particular may benefit from capturing ”future” stories, because it can be difficult for them to understand and imagine how their current choices or decisions influence their future selves, such as described by Hershfield’s idea of future self-continuity [45, 46, 48, 90]. Therefore, a potential kidney transplant journey tool should allow stakeholders to capture stories during different periods of their kidney transplant journey including future stories. Future stories could capture different topics, such as youths’ dreams and aspirations about ”when they grow up” or more mundane topics, such as successfully scheduling their own medical appointments or handling their prescriptions independently.

Importantly, future stories could also capture the fear and worry patients and/or caregivers have about growing up with kidney disease and living with a kidney transplant. A collection of current and future stories allow for collaborative reflection, leading to the development of strategies to help youth achieve their best possible future outcomes based on their current state. For example, should a future story identify the youth taking a more active role in their self-management, yet currently still rely on their caregivers, a collaborative discussion can focus on concrete steps and actions which would help the youth gain more independent skills. In addition, if a future story captures undesirable events or fears, the team can reflect on and develop prevention strategies. Therefore a future kidney transplant journey tool to capture patients and caregivers written or audio recorded transplant journey stories should support self-reflection and promote collaboration between youth patients, caregivers, and clinicians to identify and address previously unrecognized barriers that interfere with their future goals and aspirations.

### 5.2 Encouraging Self-Efficacy

While sharing stories across stakeholders has the potential to support collaboration with others, interestingly, in our findings we noticed that the process of sharing their kidney transplant experiences actually supported their own reflection, leading to new insights about their kidney transplant journey and facilitated self-efficacy. As youth living with a chronic illness grow up, most eventually become independent, and responsible for managing their health and other life needs [7, 9, 106]. This transition requires that youth engage in decision making and self-management, a frequent topic explored by clinical, CSCW, and HCI researchers alike [10, 21, 53, 55, 121, 122]. Decision making and self-management are two examples of skills that affect youth’s self-efficacy, i.e. youths’ belief in themselves to act in ways necessary to reach their goals. Limited self-efficacy prevents their drive to pursue future independence and autonomy. Yet, for many youth, it can be challenging to see their own growth and progress. Therefore capturing stories over time which highlight their individual successes, especially when they have overcome barriers, can facilitate perceived self-efficacy, which in turn facilitates self-management [51].

A key challenge to self-efficacy for youth living with a chronic illness is when their healthcare and non-healthcare life needs conflict. It can be difficult to decipher how health and life needs intertwine and affect one another throughout the course of an individuals journey. The rich stories describing the transplant journeys from youth and caregivers revealed many barriers between their healthcare and life needs and how these barriers interfered with self-efficacy. Yet, the stories also demonstrated how these youth and caregivers are constantly striving to harmonize their life and healthcare needs and overcome barriers, but it can be difficult to identify these different needs and conflicts.

As a first step, capturing stores of conflict provides an opportunity to consider all their healthcare and non-healthcare life needs, within the same context, and therefore provides a more holistic understanding of their transplant journey. To facilitate this, participants need to organize their stories into relevant topics, especially those which are frequently known to highlight conflict. In addition, we suggest that participants have the ability to tag their stories by the categories we identified in our findings to provide additional context. By tagging their stories with these different categories, youth and caregivers can more easily seek targeted opportunities for collaborative problem solving (i.e. overcoming fear or building confidence). Providing youth with a better understanding of their healthcare and life needs may help them feel more confident in understanding the barriers they experience between their needs and build self-efficacy.

### 5.3 Visualizing Information to Augment Stories

**Fig. 2.**
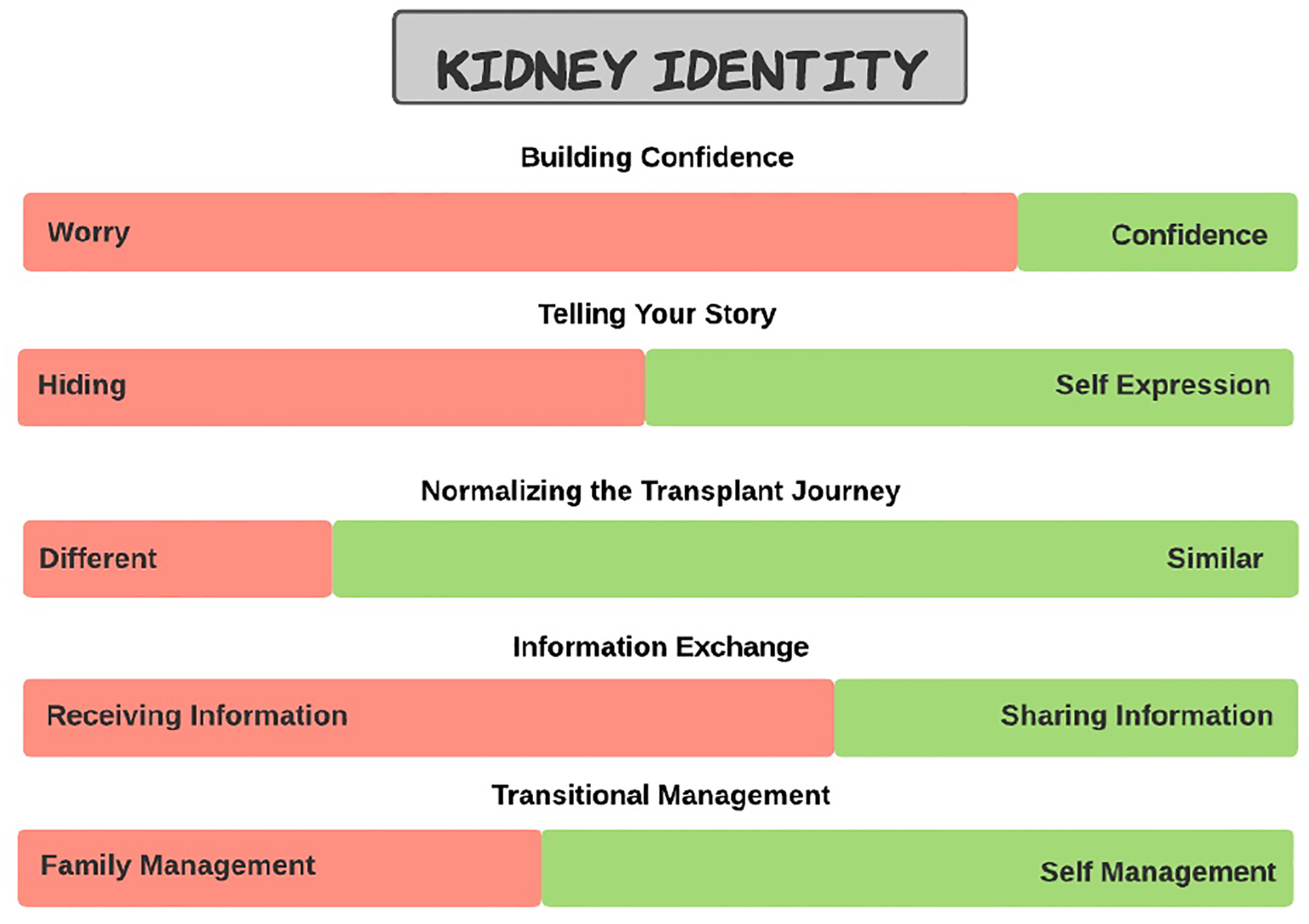
This figure depicts an idea for a design feature that could be a part of a future Kidney Journey tool. The depicted design feature is an image of the five dimensions of Dunbar et al.’s Kidney Identity framework [22] as horizontal slider bar scales. Each of the five slide bars are one of the five dimensions and the two sides of the slider bars are the two sides of the dimensions.

We found that using an existing framework to probe participants about specific parts of their transplant journey gave them structure to tell us rich stories about their experiences. Expanding upon the success of using this existing framework to probe participants about their experiences, Dunbar et al’s framework could also be used to capture a snapshot of their experiences in a different medium. Each of the dimensions resonated with participants, however, participants may not always have the time or energy to tell rich stories. To supplement the narrative stories of the transplant journey, visual features could be incorporated that capture the self-reported state of an individual within each of the five ”Kidney Identity” dimensions. Other research has shown that such health visualizations can help participants understand their health outcomes [98, 107, 115]. By moving a slider bar between the two tensions for each dimension (e.g. Building confidence: were they felling more worried or more confident), provides another way to capture the story (see Figure 2). A participant could choose to capture their ”Kidney Identity” slider bars either alongside their story or as a separate source of information on its own. The juxtaposition of the qualitative components of the narrative story with the quantitative elements of the visualization, provides new opportunities for reflection, especially when compared over time. Even though stories provide invaluable contextualized information into individuals’ transplant journeys, we recognize writing or audio recording stories can be time consuming. Giving participants the option to capture their dimensions in another format either alongside their stories or on their own could be a way to instead provide a quick snapshot of moments during their transplant journey. At an individual level, participants could benefit from capturing their stories, but may also benefit from quicker snapshots of their journey over time. The proposed health visualization expands on Dunbar et al’s framework by providing participants with another medium to capture information about their transplant journey over time, facilitating another way to help youth, caregivers, and their care team to collaboratively identify barriers, support reflection, and promote self-efficacy for youth kidney transplant patients.

### 5.4 Sharing Stories

Even though our study shows numerous individual benefits to developing a tool for youth participants to capture their kidney transplant journey stories, we are still determining whether and how it may benefit participants to see or listen to other kidney transplant community members’ stories. In our findings, we consistently heard from both youth and their caregivers that they have rarely or never interacted with other youth kidney transplant patients. Individuals with chronic illnesses often lack interaction with others in their own chronic illness communities and thus many adults with chronic illnesses turn to online communities [97]. Individuals join these different types of online communities (e.g., social media or vlogs) for a variety of reasons including, but not limited to using them as a form of support for chronic illness management or to engage with others who have similar experiences as their own [31, 58, 60, 64, 79]. Online communities provide many benefits to individuals who are on them, such as being able to share stories [56], but these online communities typically lack structure with how information is distributed making it difficult for individuals to find content they are interested in or build relevant networks of people with similar experiences [14, 88]. Also, these online communities are usually developed with adults in mind and lack appropriate moderation for youth.

Giving youth access to other transplant patients stories may help them feel like a part of a kidney transplant community, which most of these individuals lack. Capturing stories from a variety of transplant patients will allow individuals to view other transplant patients’ stories and recognize similarities or differences to their own story. Recognizing these similarities and differences may benefit these youth by making them feel less alone or helping them normalize their experiences. Since many of these youth lack the ability to interact with other youth with their same illness, they may not have a baseline understanding of what others go through. Accessing others stories may provide youth with interesting healthcare and life related information that can help youth normalize and compare their own experiences against others. In addition, it might help them identify previously unrecognized problems or challenges that they have adapted to, by identifying differences with other transplant recipients. Lastly, there is an opportunity to learn from other youth, specifically by seeing how others have approached and overcome similar barriers or challenges.

Although being able to compare their experiences may be beneficial for youth, such as being able to normalize or learn from others experiences, it also runs the risk of the youth negatively comparing themselves to others and creating unrealistic expectations [3, 33]. We see potential in allowing youth to have access to other transplant patients anonymous stories in a future kidney transplant journey tool, but feel that this needs further exploration prior to implementation within a new tool.

### 5.5 Limitations and Future Work

Our study has several limitations which need to be considered. All of our participants were from a single institution and most likely reflect the population of that area. A majority of our participants were Caucasian and English-speaking; future work should engage more diverse populations. We had a wide age-range of youth who participated (7 – 22 years of age). Although we included younger participants because their perspectives are not typically reflected in this type of work, we recognize participants’ developmental stages and other age-based considerations could affect their perspectives. Also, most of our participants did not recently have their kidney transplant, thus recall bias from participants could affect their perceptions. Even though our findings support the value of stories, which provide rich contextualized information, we recognize that capturing stories can be burdensome for individuals compared to other types of information. Using the Dunbar et. al framework influenced whether and how youth could reflect on their experiences. Other frameworks might encourage participants to reflect on different aspects of their experiences. Although other frameworks and similar literature do touch on aspects of the chronic illness journey, we posit the rich accounts of experiences we captured stemmed from the holistic nature of the Dunbar et al framework. Our future work plans to include these findings to design an initial *KJ tool* prototype and explore its potential usefulness with youth transplant patients.

## 6 CONCLUSION

Youth with severe chronic illnesses, their caregivers, and their clinical care team deal with many complexities throughout a youth’s chronic illness. Therefore, capturing these complexities may help create a more comprehensive picture of a youth’s chronic illness journey. In this study, we did semi-structured interviews with 11 pediatric kidney transplant patients and 12 caregivers. Our work demonstrates how the framework successfully allowed our participants to tell their kidney transplant journey experiences. The five dimensions of the Kidney Identity framework supported participants as they told rich stories of their experiences, and more importantly, highlighting the conflicts and synergies between their health and non-health life needs. We illustrate that capturing the stories of youth kidney transplant patients and their caregivers, supports stakeholders in reflecting on their experiences and collaboratively identifying and communicating health and life barriers. We hope our study can inform future work to help youth with severe chronic illnesses capture a more comprehensive view of their journeys, identify barriers towards harmonizing their healthcare and non-healthcare needs, and ultimately help them live the lives they want to live.

## Figures and Tables

**Table 1.** Pediatric Transplant Patients and Caregiver Demographics

Demographics	*Patients (n = 11)*	*Caregivers (n=12)*
Age (In Years)	7 – 13 = 6 14 – 18 = 4 19 – 22 = 1	25 – 34 = 1 35 – 44 = 4 45 – 55 = 4 55+ = 2 Not Reported = 1
Gender	Man/Boy = 9 Woman/Girl = 2 Transgender = 1 *One participant selected two genders*	Woman/Girl = 11 Not Reported = 1
Race	White = 8 Chinese = 1 Some Other Race = 1 Not Reported = 1	White = 9 Chinese = 1 Some Other Race = 1 Not Reported = 1
Ethnicity	Not of Hispanic, Latino, or Spanish Origin = 8 Mexican, Mexican American, or Chicano = 2 Decline to State = 1	Not of Hispanic, Latino, or Spanish Origin = 10 Mexican, Mexican American, or Chicano = 1 Not Reported = 1

**Table 2.** Story Categories Captured from the Kidney Identity Framework Dimensions

Kidney Identity Framework Dimensions	Story Categories
Telling People About Your Kidney Transplant Journey	Stories about Barriers and Benefits to Sharing About Your Journey Stories about Community
Exchanging Information Throughout the Transplant Journey	Stories about Lack of Interaction with Other Transplant Families Stories about Building Self-Efficacy
Transitional Management Throughout the Transplant Journey	Stories about Responsibility and Planning for the Future Stories about Successes and Challenges of Transitioning
Building Confidence Throughout the Transplant Journey	Stories about Fear Stories about the Road to Confidence
Normalizing the Transplant Journey	Stories about Removing Healthcare Burdens and Reconnecting with Your Community Stories about Accepting Differences and Establishing a “New Normal”

## Box

CCS Concepts: **• Human-centered computing** → **Empirical studies in collaborative and social computing**; • **Applied computing**→**Health informatics**; **Consumer health**.
